# Comparison of antimicrobial susceptibility of *Glaesserella parasuis* from different pig production systems in Taiwan between 2015 and 2020

**DOI:** 10.1186/s40813-025-00427-8

**Published:** 2025-03-18

**Authors:** Wei-Hao Lin, Zhu-Wei Liou, Szu-Min Lin, Cheng-Yao Yang, Chuen-Fu Lin, Yung-Fu Chang, Chao-Nan Lin, Ming-Tang Chiou

**Affiliations:** 1 Animal Disease Diagnostic Center, College of Veterinary Medicine, National Pingtung University of Science and Technology, Pingtung, 912301 Taiwan; 2 Department of Veterinary Medicine, College of Veterinary Medicine, National Pingtung University of Science and Technology, Pingtung, 912301 Taiwan; 3 Sustainable Swine Research Center, National Pingtung University of Science and Technology, Pingtung, 912301 Taiwan; 4 Graduate Institute of Veterinary Pathobiology, College of Veterinary Medicine, National Chung Hsing University, Taichung, 402202 Taiwan; 5 Department of Population Medicine and Diagnostic Sciences, College of Veterinary Medicine, Cornell University, Ithaca, NY 14853 USA

**Keywords:** Antimicrobial susceptibility, *Glaesserella parasuis*, Glässer’s disease, Minimal inhibitory concentration, Pig

## Abstract

**Background:**

Glässer’s disease, caused by *Glaesserella parasuis* (*G. parasuis*), is a widespread bacterial infection in swine that leads to significant economic losses. *G. parasuis*, a member of the normal microbiota within the *Pasteurellaceae* family, exhibits horizontal resistance gene exchange and intracellular invasion capabilities, increasing the risk of developing resistant isolates. Accurate antimicrobial therapy is essential for controlling Glässer’s disease. The production systems for exotic crossbred pigs and Taiwan black pigs differ considerably. To inform Glässer disease control and monitor antimicrobial resistance, we assessed the antimicrobial susceptibilities of *G. parasuis* isolates, analyzed them using normalized resistance interpretation (NRI), and compared findings between the two production systems.

**Results:**

A total of 154 *G. parasuis* isolates from 106 exotic crossbred pig herds and 48 Taiwan black pig herds were tested against 16 antimicrobial agents between 2015 and 2020. Due to the absence of specific breakpoints for *G. parasuis*, NRI was utilized to define non-wild-type (non-WT) populations based on minimum inhibitory concentration (MIC) distributions. Non-WT subpopulations of isolates for amoxicillin, ampicillin, ceftiofur, gentamicin, kanamycin, and tiamulin were observed. The highest MIC_90_ (the concentration at which 90% of isolates were inhibited) was > 256 µg/mL for several antimicrobials, including gentamicin, kanamycin, lincomycin, lincospectin, spectinomycin, and tylosin. In contrast, the lowest MIC_90_ was observed for ceftiofur (0.5 µg/mL). The MIC values for cephalothin were significantly higher in exotic crossbred pigs than in Taiwan black pigs (*p* = 0.0016). Conversely, MIC values for florfenicol were significantly higher in Taiwan black pigs than in exotic crossbred pigs (*p* = 0.003).

**Conclusions:**

This study provides the susceptibility profile of *G. parasuis* isolates for both exotic crossbred pigs and Taiwan black pigs in Taiwan and highlights potential antimicrobial resistance for aminocyclitol, aminoglycosides, beta-lactams, lincosamides, macrolides, and pleuromulin. Ceftiofur, cephalothin, doxycycline, and florfenicol could be most suitable for treating early-stage Glässer’s disease. Nonetheless, increased attention should be paid to the responsible use of antimicrobials in light of the growing threat of antimicrobial resistance.

**Supplementary Information:**

The online version contains supplementary material available at 10.1186/s40813-025-00427-8.

## Background

*Glaesserella parasuis* (*G. parasuis*) is a Gram-negative bacterial pathogen responsible for Glässer’s disease [1]. Glässer’s disease causes meningitis, serositis, arthritis, pneumonia, and reduced growth performance in pigs, leading to substantial economic losses [2]. Clinical signs of Glässer’s disease include fever, abdominal breathing, coughing, lameness, paddling, septicemia, and sudden death [3]. *G. parasuis* is a resident microbiota typically present in the upper respiratory tract of pigs [4]. *G. parasuis* is primarily transmitted through direct contact with carriers or diseased pigs, especially by purchasing pigs from different sources [1, 5]. The virulence of *G. parasuis* varies significantly, as it serves as an opportunistic agent while also being a primary pathogen responsible for swine diseases globally [5, 6].

The exotic crossbred pigs of Western breeds, including Landrace, Yorkshire, and Duroc, are the primary source of pork in Taiwan. Taiwan black pigs retain a market share in Taiwan due to local traits such as higher intramuscular fat content [7]. Due to their slower growth rate and lower feed efficiency (approximately 12–15 months), the production system of Taiwan black pig farms is more traditional and operates on a smaller scale compared to that of exotic crossbred pigs [8]. Because exotic crossbred pigs and Taiwan black pigs are not raised on the same farm, the direct transmission of *G. parasuis* between these two populations is likely rare.

*G. parasuis* bacterin vaccines have been widely used to combat Glässer’s disease. However, most vaccines do not include all the prevalent serovars found in different countries [9]. Additionally, the efficacy of *G. parasuis* bacterin vaccines varies depending on the virulence factor, serotype, and antigenicity of the vaccine strains [10–13]. In Taiwan, the prevalent serovars are serovars 4, 5, 13, and non-typable isolates [14, 15], but the only available commercial vaccine covers serovar 5 (Porcilis Glässer, MSD).

Antimicrobial therapy remains an effective strategy for controlling and preventing *G. parasuis,* and selecting appropriate antimicrobials for affected animals in the early stages of Glässer’s disease is crucial [1, 5]. In Taiwan, various antimicrobials, including penams, cephalosporins, tetracyclines, and macrolides, have been widely used to combat respiratory pathogens. As a normal component of the microbiota, *G. parasuis* may be more prone to developing antimicrobial resistance (AMR). Determining antimicrobial susceptibility profiles is essential for selecting appropriate antimicrobial agents to treat Glässer’s disease. This study aims to assess the antimicrobial susceptibility profile of Taiwanese *G. parasuis* and compare the distribution of minimum inhibitory concentrations (MICs) between isolates from exotic crossbred pigs and Taiwan black pigs.

## Methods

### Bacterial isolate collection and identification

A total of 154 *G. parasuis* isolates, comprising 106 from exotic crossbred pig herds and 48 from Taiwan black pig herds, were collected between 2015 and 2020. The isolates were obtained from lesions of pigs diagnosed with Glässer’s disease (Additional file 1). Clinical cases were examined and diagnosed at the Animal Disease Diagnostic Center, National Pingtung University of Science and Technology. The bacterial samples were cultured in a 5% carbon dioxide environment and a temperature of 37 ℃ for 24 h. Chocolate agar was used as the culture substrate because *G. parasuis* is a fastidious organism that requires specific culture medium components for growth.

The KAPA2G Fast HotStart ReadyMix (Kapa Biosystems, Roche, Basel, Switzerland) and the ProFlex PCR System (Applied Biosystems, Carlsbad, CA, USA) were used for the polymerase chain reaction to identify *G. parasuis* isolates [14, 16]. The thermocycling conditions were as follows: initial denaturation at 94 °C for 5 min, followed by 30 cycles of denaturation at 94 °C for 30 s, annealing at 58 °C for 30 s, and extension at 72 °C for 1 min, with a final extension at 72 °C for 5 min. The primer sequences used (at 0.04 mM concentration) were 5′-ACAACCTGCAAGTACTTATCGGGAT-3′ (forward) and 5′-TAGCCTCCTGTCTGATATTCCCACG-3′ (reverse).

### Antimicrobial sensitivity test

The antimicrobial sensitivity of *G. parasuis* was assessed using a broth microdilution assay. *G. parasuis* isolates were cultured in cation-adjusted Mueller–Hinton broth (BD Difco, Sparks, MD, USA) supplemented with 1% chicken serum (Gibco, Grand Island, NY, USA) and 0.0025% beta-nicotinamide adenine dinucleotide hydrate (Sigma-Aldrich, St. Louis, MO, USA) and incubated in a 5% CO_2_ environment for 24 h at 37 °C, as CLSI-approved veterinary fastidious medium is not suitable for all *G. parasuis* isolates [17]. The susceptibility of the isolates was tested against 16 antimicrobials using 96-well optical bottom plates (Nunc™, Roskilde, Denmark), including amoxicillin, ampicillin, ceftiofur, cephalothin, colistin, doxycycline, enrofloxacin, florfenicol, gentamicin, kanamycin, lincomycin, lincospectin (1:2), spectinomycin, tiamulin, tilmicosin and tylosin. All antimicrobial agent stock solutions had concentrations at least ten times higher than the highest concentration to be tested according to the potency information provided by the manufacturer. Sterile distilled water or the recommended solvent were used for preparing all solutions, which were filter-sterilized using 0.22-μm pore size cellulose-acetate filters (Millipore, Germany). Inoculum quantification was conducted by measuring the optical density at 600 nm (OD_600_) using a UV–VIS spectrophotometer (U-2900, Hitachi, Japan). The 96-well optical bottom plate format was used as it permits testing ten different concentrations of each antimicrobial agent along with one growth control (broth with bacterial inoculum, no antimicrobial) and one sterility control (broth only). Details regarding the concentration ranges of antimicrobial agents tested are provided in Table 1.

**Table 1:** Minimum inhibitory concentration (MIC) distribution of 154 *G. parasuis* isolates in Taiwan

Antimicrobial	Number of isolates with MIC (µg/mL)																		MIC_50_	MIC_90_
	0.004	0.008	0.015	0.03	0.06	0.12	0.25	0.5	1	2	4	8	16	32	64	128	256	> 256		
Amoxicillin				5^a^	7	31	37	15	12^b^	4	10	7	4	*22*					0.25	> 16
Crossbred				2^a^	6	20	25	11	7	2	8	6	1	*18*					0.25	> 16
Black pig				3^a^	1	11	12	4	5	2	2	1	3	*4*					0.25	16
Ampicillin				2^a^	3	7	18	42	26	8	5	8^b^	4	*31*					1	> 16
Crossbred				1^a^	2	5	11	25	18	8	3	5	4	*24*					1	> 16
Black pig				1^a^	1	2	7	17	8	0	2	3	0	*7*					0.5	> 16
Ceftiofur			45^a^	25	12^b^	22	34	4	2	2	0	1	*7*						0.06	0.5
Crossbred			32^a^	16	8	15	23	3	0	1	0	1	*7*						0.06	0.5
Black pig			13^a^	9	4	7	11	1	2	1	0	0							0.06	0.25
Cephalothin			2^a^	5	8	9	7	22	32	26	19	13	*11*		^b^				1	8
Crossbred			2^a^	3	1	6	4	11	23	22	15	11	*8*						2	8
Black pig			0	2	7	3	3	11	9	4	4	2	*3*						0.5	8
Colistin	0	0	0	0	0	1	10	56	50	27^b^	*10*								1	2
Crossbred	0	0	0	0	0	1	7	33	36	21	*8*								1	2
Black pig	0	0	0	0	0	0	3	23	14	6	*2*								0.5	2
Doxycycline			0	0	1	0	2	7	50	69	24	0^b^	*1*						2	4
Crossbred			0	0	1	0	0	6	30	48	21	0							2	4
Black pig			0	0	0	0	2	1	20	21	3	0	*1*						2	2
Enrofloxacin							7^a^	1	5	33	56	21	22	5^b^	1	0	*3*		4	16
Crossbred							5^a^	1	4	21	43	13	12	4	1	0	*2*		4	16
Black pig							2^a^	0	1	12	13	8	10	1	0	0	*1*		4	16
Florfenicol					0	1	0	26	31	43	34	16	0^b^	0	*3*				2	8
Crossbred					0	1	0	24	19	33	20	8	0	0	*1*				2	4
Black pig					0	0	0	2	12	10	14	8	0	0	*2*				2	8
Gentamicin								5^a^	8	40	42	6	2^b^	4	4	4	11	*28*	4	> 256
Crossbred								3^a^	6	27	27	4	1	3	4	3	5	*23*	4	> 256
Black pig								2^a^	2	13	15	2	1	1	0	1	6	*5*	4	> 256
Kanamycin								0	2	4	13	63	14	0^b^	1	2	5	*50*	8	> 256
Crossbred								0	2	4	8	42	9	0	0	1	3	*37*	8	> 256
Black pig								0	0	0	5	21	5	0	1	1	2	*13*	8	> 256
Lincomycin								0	0	1	2	10	9	1	4^b^	5	5	*117*	> 256	> 256
Crossbred								0	0	1	2	9	7	1	1	4	3	*78*	> 256	> 256
Black pig								0	0	0	0	1	2	0	3	1	2	*39*	> 256	> 256
Lincospectin (1:2)								0	1	2	6	11	6	10	13	14^b^	12	*79*	> 256	> 256
Crossbred								0	1	2	5	10	5	6	9	10	6	*52*	> 256	> 256
Black pig								0	0	0	1	1	1	4	4	4	6	*27*	> 256	> 256
Spectinomycin								0	0	0	3	10	10	4	7^b^	15	18	*87*	> 256	> 256
Crossbred								0	0	0	3	7	8	2	6	13	11	*56*	> 256	> 256
Black pig								0	0	0	0	3	2	2	1	2	7	*31*	> 256	> 256
Tiamulin								0	8	20	30	26	22	17	10^b^	5	4	*12*	8	256
Crossbred								0	5	17	20	21	18	5	4	4	2	*10*	8	256
Black pig								0	3	3	10	5	4	12	6	1	2	*2*	16	128
Tilmicosin						0	1	7	4	7	5	5	16	21	28	*60*			64	> 64
Crossbred						0	1	5	4	5	5	3	11	13	20	*39*			64	> 64
Black pig						0	0	2	0	2	0	2	5	8	8	*21*			64	> 64
Tylosin								2^a^	3	3	5	6	8	12	26	34	34	*21*	128	> 256
Crossbred								2^a^	1	2	5	5	6	8	18	24	23	*12*	128	> 256
Black pig								0	2	1	0	1	2	4	8	10	11	*9*	128	> 256

Roman indicate the tested concentrations of antimicrobials

Numbers in italics indicate the minimal inhibitory concentration (MIC) values higher than the highest concentration in the tested range

^a^The number of isolates with MIC values equal to or lower than the tested concentration range

^b^The wild-type cut-off (CO_WT_) calculated by normalized resistance interpretation

*Actinobacillus pleuropneumoniae* strain ATCC 27090, *Escherichia coli* (*E. coli*) strain ATCC 25922, and *Enterococcus faecalis* strain ATCC 29212 were used for every batch of antimicrobial susceptibility testing to control for factors related to plate preparation, reagent quality, and environmental conditions [18, 19]. Because the broth containing *G. parasuis* was relatively clear, OD_600_ values of 96-well plate samples were measured after incubation to assist in interpreting possible growth patterns in MIC microtiter plates [18]. The OD_600_ values of sterility control were lower than 0.04, and the OD_600_ values of growth controls varied from 0.05 to 0.13 based on the *G. parasuis* isolates.

### Data analysis

Binary logarithms of the MIC values (mg/L) were calculated. Because specific veterinary guidelines for determining *G. parasuis* antimicrobial resistance are lacking, the normalized resistance interpretation (NRI) method was used to distinguish wild-type (WT) and non-wild-type (non-WT) populations, which includes groups with acquired or mutational resistance [20, 21]. All MIC susceptibility measure data were recorded as log_2_ μg/mL values. The NRI calculation for MIC data was performed using an automated Excel spreadsheet downloaded from http://www.bioscand.se/nri/. This spreadsheet was also used to calculate the means and standard deviations of the normalized distributions (SD_MIC_). An SD_MIC_ value greater than or equal to 1.2 log_2_ μg/mL was considered indicative of an abnormal standard deviation. The wild-type cutoff (CO_WT_) values were determined by adding 2 SD_MIC_ to the calculated means [20, 21]. All statistical data were analyzed using Prism 10.3.1 (GraphPad Software Inc., La Jolla, CA, USA). The MIC distributions between exotic crossbred pigs and Taiwan black pigs were compared using the Mann-Whitney U test, as they did not follow a continuous probability distribution and failed the normality assumption as assessed using the Shapiro–Wilk test. Statistical significance was set at *p* < 0.05.

## Results

### MIC value distribution and epidemiological breakpoint

A total of 154 *G. parasuis* isolates were collected from 106 exotic crossbred pig herds and 48 Taiwan black pig herds with Glässer’s disease. The MIC values for the *G. parasuis* isolates are presented in Table 1. Graphs representing the MIC distributions of all 16 antimicrobials are shown in Additional file 2. The MIC distribution patterns, wild-type cutoff (CO_WT_), standard deviations of the normalized distributions (SD_MIC_), and percentages of WT and non-WT isolates are listed in Table 2.

**Table 2:** Minimum inhibitory concentration (MIC) distribution patterns, wild-type cutoff (CO_WT_), and frequencies of wild-type (WT) and non-wild type (non-WT) isolates

Class (subclass)	Antimicrobial	MIC distribution pattern	Breakpoint^a^ (µg/mL)	CO_WT_ (µg/mL)	SD_MIC_ (log_2_ µg/mL)	WT		Non-WT	
						n	%	n	%
*Beta lactams*									
(Penams)	Amoxicillin	Multi-modal	2	1	1.23	107	69.5	47	30.5
	Ampicillin	Multi-modal	2	8	1.51^b^	119	77.3	35	22.7
(Cephalosporins)	Ceftiofur	Multi-modal	8	0.06	0.6	82	53.2	72	46.8
	Cephalothin	Unknown	N/A	64	2.94^b^	154	100	0	0
Polymyxins	Colistin	Unimodal	N/A	2	0.84	144	93.5	10	6.5
Tetracyclines	Doxycycline	Unimodal	N/A	8	0.91	153	99.4	1	0.6
Fluoroquinolones	Enrofloxacin	Unimodal	1	32	1.62^b^	150	97.4	4	2.6
Amphenicols	Florfenicol	Unimodal	8	16	1.56^b^	151	98.1	3	1.9
Aminoglycosides	Gentamicin	Multi-modal	N/A	16	1.11	103	66.9	51	33.1
	Kanamycin	Multi-modal	N/A	32	0.86	96	62.3	58	37.7
Aminocyclitol	Spectinomycin	Unknown	N/A	64	1.07	34	22.1	120	77.9
Lincosamides	Lincomycin	Unknown	N/A	64	1.07	27	17.5	127	82.5
	Lincospectin (1:2)	Unknown	N/A	128	1.48^b^	63	40.9	91	59.1
Pleuromutilin	Tiamulin	Multi-modal	32	64	1.41^b^	133	86.4	21	13.6
Macrolides	Tilmicosin	Unknown	32	32,768	3.92^b^	154	100	0	0
	Tylosin	Unknown	N/A	32,768	3.37^b^	154	100	0	0

^a^Breakpoint for swine *Pasteurella multocida* and *Actinobacillus pleuropneumoniae* published in CLSI VET01S ED7:2024

^b^standard deviation of the CO_WT_ (SD_MIC_) calculation was abnormal

Using CO_WT_ values derived from the NRI method, high WT population percentages were observed for colistin (93.5%) and doxycycline (99.4%) with normal SD_MIC_ values. Conversely, 82.5% and 77.9% of the isolates were categorized as non-WT for lincomycin and spectinomycin, respectively. The SD_MIC_ values for ampicillin, cephalothin, enrofloxacin, florfenicol, lincospectin (1:2), tiamulin, tilmicosin, and tylosin were higher than the upper limit (1.2 log_2_ µg/mL) and were classified as abnormal.

Unimodal MIC distributions were observed for colistin, doxycycline, enrofloxacin, and florfenicol. For these antimicrobials, a few isolates were still defined as non-WT populations by the NRI. Multimodal MIC distributions were observed for amoxicillin, ampicillin, ceftiofur, gentamicin, kanamycin, and tiamulin. Because more than 50–75% of the isolates exhibited resistance to the maximal antimicrobial dilution tested for spectinomycin, lincomycin, and lincospectin (1:2), their distribution patterns could not be determined. The undefined distribution patterns were due to half-bell-shaped clustering observed for tilmicosin and tylosin.

### Antimicrobial susceptibility comparison between crossbred and black pig herds

**Fig. 1:**
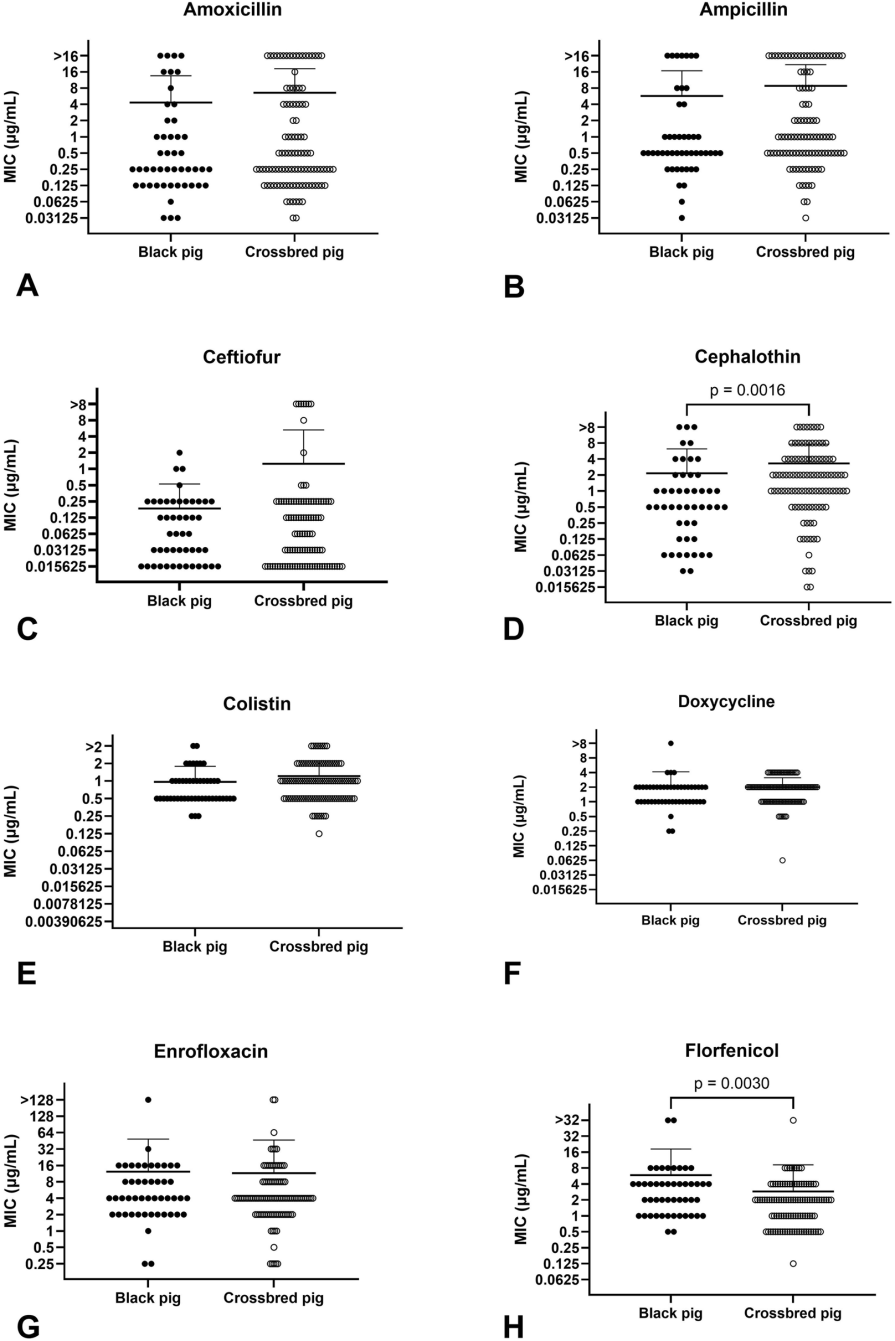
Minimum inhibitory concentration (MIC) distributions of *G. parasuis* isolates for beta-lactams, polymyxins, tetracyclines, fluoroquinolones, and amphenicols. MIC distributions for amoxicillin (**A**), ampicillin (**B**), ceftiofur (**C**), cephalothin (**D**), colistin (**E**), doxycycline (**F**), enrofloxacin (**G**), and florfenicol (**H**). Data were analyzed using the Mann-Whitney U test. Statistical significance was set at *p* < 0.05

**Fig. 2:**
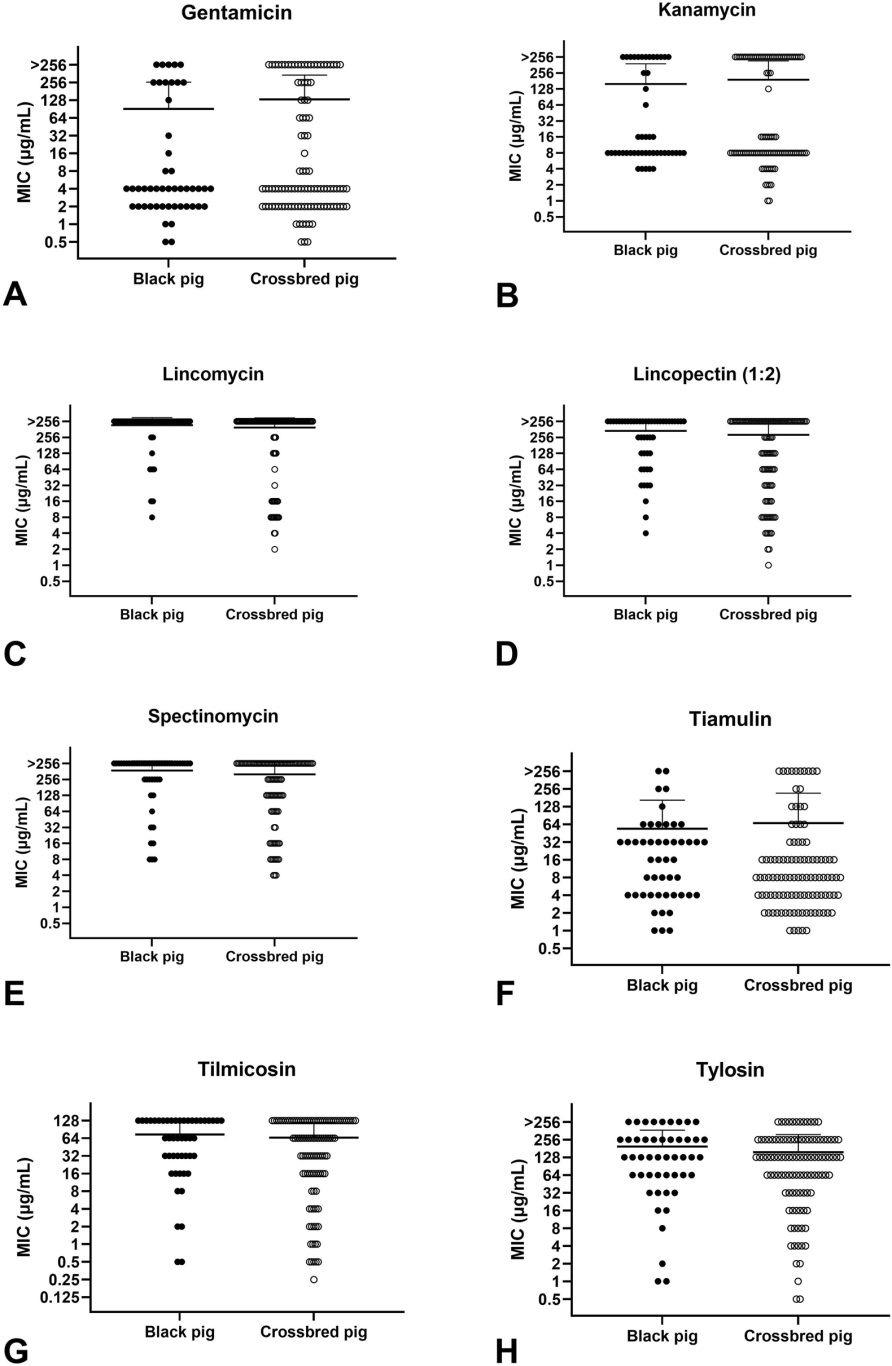
Minimum inhibitory concentration (MIC) distributions of *G. parasuis* for aminoglycosides, lincosamides, aminocyclitols, tiamulin and macrolides. MIC distributions for gentamicin (**A**), kanamycin (**B**), lincomycin (**C**), lincospectin (1:2) (**D**), spectinomycin (**E**), tiamulin (**F**), tilmicosin (**G**), and tylosin (**H**). Data were analyzed using the Mann-Whitney U test. Statistical significance was set at *p* < 0.05

The MIC values for *G. parasuis* isolates from 106 exotic crossbred pig herds and 48 Taiwan black pig herds for all antimicrobials are shown in Figs. 1 and 2. No significant differences in MIC values were found between crossbred and Taiwan black pigs for any antimicrobial, except cephalothin and florfenicol. The geometric mean MIC for cephalothin was significantly higher in exotic crossbred pigs (1.38 ± 4.75) compared to Taiwan black pigs (0.61 ± 5.33) (*p* = 0.0016). Conversely, the geometric mean MIC for florfenicol was significantly higher in Taiwan black pigs (2.83 ± 2.8) compared to exotic crossbred pigs (1.64 ± 2.6) (*p* = 0.003).

## Discussion

*G. parasuis* is a common pathogen affecting swine production worldwide [22, 23]. Prompt selection of effective antimicrobials is crucial for managing Glässer’s disease [24]. Without appropriate antimicrobial therapy, fibrinous lesions may become fibrotic, leading to chronic damage and reduced growth rate. To the best of our knowledge, this is the first study in Taiwan investigating the antimicrobial susceptibility of *G. parasuis* isolated from exotic crossbred and Taiwan black pigs. *G. parasuis* is a member of the *Pasteurellaceae* family, which includes the *Pasteurella*, *Actinobacillus,* and *Haemophilus* species. The exchange of resistance genes between members of the *Pasteurellaceae* family is primarily facilitated by horizontally transferred plasmids and transposons [25, 26]. As a commensal organism in the upper respiratory tract of swine [4], *G. parasuis* isolates are exposed to various antimicrobial treatments, increasing the risk of developing AMR. *G. parasuis* can invade macrophages, epithelial cells, and endothelial cells [5, 27–29], which may increase the risk of selecting resistant isolates. AMR in *G. parasuis* may serve as a reservoir for monitoring resistance patterns in swine.

The proportion of resistant isolates in this study could not be determined due to the lack of established breakpoints specific to *G. parasuis*. The NRI offers an objective method for analyzing MICs and determining CO_WT_ distribution and has been applied to many bacteria, including *Staphylococcus aureus*, *E. coli*, and *Klebsiella pneumoniae*, as an alternative to traditional breakpoints [20, 21]. Recently, the development of pharmacokinetic/pharmacodynamic (PK/PD) models for ceftiofur sodium and enrofloxacin against *G. parasuis* in pigs has been reported [30, 31]. By integrating more PK/PD models with additional available data, such as epidemiologic cut-off and clinical cut-off data, specific susceptible breakpoints can be established in the future.

The finding of multi-modal MIC distributions and the presence of non-WT isolates based on CO_WT_ indicates the AMR of amoxicillin, ampicillin, ceftiofur, gentamicin, kanamycin, and tiamulin. Penams are probably the most widely used antibiotics for the treatment and control of bacterial infections in pigs [32], and tiamulin is frequently used as a feed additive to control *Mycoplasma* infections in swine [33]. Compared to those in Brazil, the Czech Republic, Germany, the United Kingdom, and Spain [17, 34–37], the high MIC_90_ values for aminoglycosides observed in Taiwan were consistent with those reported in China [38]. This may be attributed to the extensive parenteral use of aminoglycosides, which are not absorbed in the gastrointestinal tract [39]. However, the NRI-derived CO_WT_ for ceftiofur (0.06 µg/mL) was much lower than the breakpoint for swine *Pasteurella multocida* and *Actinobacillus pleuropneumoniae* (8 µg/mL). This may have been underestimated, as most isolates exhibited MIC values at or below the lowest tested concentration. Nonetheless, there were still some isolates with MIC values for ceftiofur higher than 8 µg/mL. The MIC_90_ for ceftiofur (0.5 μg/mL) was lower than that of penams and comparable to reports from Brazil, Germany, and Taiwan [15, 36, 40]. The restriction on the antimicrobial administration route may explain the observed patterns, as cephalosporins are only permitted for treating sick pigs via intramuscular injection in Taiwan.

If the SD_MIC_ is abnormally high (SD_MIC_ ≥ 1.2 log_2_ μg/mL), the CO_WT_ should either not be used or should be interpreted in conjunction with other values [41]. The high genetic diversity of *G. parasuis* isolates [42, 43] may contribute to an increased SD_MIC_. In our study, the CO_WT_ with abnormal SD_MIC_ differed from the breakpoints for swine *Pasteurella multocida* and *Actinobacillus pleuropneumoniae*. Although the use of breakpoints for other bacteria to claim resistant isolates may not be accepted, it could still be a point of discussion since *G. parasuis* is also a swine respiratory bacterial pathogen in the *Pasteurellaceae* family. The broad MIC distributions suggest putative AMR for cephalothin, tilmicosin, and tylosin, even though non-WT isolates for these antimicrobials were not identified using NRI calculations. The MIC values for most isolates of lincomycin, lincospectin (1:2), and spectinomycin were higher than the highest tested concentration (256 μg/mL). These results suggest widespread resistance to lincomycin and spectinomycin, which have been commonly used for decades in Taiwan. Notably, almost all porcine *Pasteurella multocida* isolates tested in a previous study in Taiwan were resistant to lincomycin and spectinomycin [44].

In contrast, unimodal MIC distributions with normal SD_MIC_ were observed for colistin and doxycycline. The MIC value distribution for colistin was comparable to that reported in Germany [17, 34, 40]. In Taiwan, colistin is primarily used for treating porcine gastrointestinal Gram-negative bacteria such as *E. coli* and *Salmonella*. Because colistin is not absorbed in the gastrointestinal tract [45], the likelihood of inducing colistin-resistant isolates is low. Rare non-WT colistin isolates may be linked to tonsillar *G. parasuis* that comes in contact with colistin in the oral cavity [40]. Inhaled colistin has been suggested as a potential treatment for human pneumonia caused by multidrug-resistant Gram-negative bacteria [46]. From a One Health perspective, colistin should be reserved as a last-resort treatment under strict regulations in pig production [47]. Despite the prohibition of colistin as a growth promoter in Taiwan since 2005, its unnecessary use without precise diagnosis still requires attention.

Duroc, Landrace, Yorkshire, and Hampshire breeds were introduced into Taiwan for crossbreeding during the 1960s. Two-way (Duroc × Landrace) and three-way (Duroc × Yorkshire × Landrace) crossbreds have become the major commercial lines in the Taiwanese pork market. Due to their slow growth rate, poor feed efficiency, and low lean meat content, the population of Taiwan black pigs has declined. However, they still account for 15.83% of the total pig population because of sustained consumer preference for their meat flavor [7]. Exotic crossbred pigs and Taiwan black pigs are managed under distinct production systems, with differences in vaccination protocols, antimicrobial strategies, and clinical management. *G. parasuis* is primarily transmitted through direct contact, while indirect transmission remains speculative [1]. To optimize Glässer’s disease control strategies and AMR monitoring in Taiwanese pig production, further investigation into the Taiwanese black pig population is necessary. Statistically significant differences in the MIC values for cephalothin and florfenicol were observed between the two populations (Fig. 1D, H). Because Taiwan black pig farms operate under more traditional production systems, they often face shortages in human resources. Individual therapy for sick pigs using intramuscular injections of cephalothin and ceftiofur is more commonly practiced on exotic crossbred pig farms. In contrast, florfenicol is preferred as a feed additive on Taiwan black pig farms.

## Conclusions

This study compared the antimicrobial susceptibility of *G. parasuis* isolates from exotic crossbred and Taiwan black pigs, identifying non-WT subpopulations for amoxicillin, ampicillin, ceftiofur, gentamicin, kanamycin, and tiamulin. High MIC_90_ was observed for aminoglycosides, aminocyclitol, lincosamides, macrolides, and pleuromutilin. Given their MIC_90_ values, ceftiofur, cephalothin, doxycycline, and florfenicol could be more suitable for treating early-stage Glässer’s disease. Effective management strategies, robust vaccination programs, and prudent antimicrobial use are essential to curbing the emergence and spread of antimicrobial resistant bacteria.

## Supplementary Information

Additional file 1.

Additional file 2.

## Data Availability

No datasets were generated or analysed during the current study.

## Abbreviations

AMR: Antimicrobial resistance
CO_WT_: Wild-type cutoff
*G. parasuis*: *Glaesserella parasuis*
MIC: Minimum inhibitory concentration
non-WT: Non-wild type
NRI: Normalized resistance interpretation
SD_MIC_: Standard deviation of normalized distributions
WT: Wild type
