# Supplementary material for: Comparison of antimicrobial susceptibility of Glaesserella parasuis from different pig production systems in Taiwan between 2015 and 2020
Source: Porcine Health Manag. 2025 Mar 18;11:15. doi: 10.1186/s40813-025-00427-8 (PMC11921561; doi:10.1186/s40813-025-00427-8)
Supplement: Supplementary file 2 — Additional file 2. [file 40813_2025_427_MOESM2_ESM.docx]

**Figure S1.** Minimum inhibitory concentration (MIC) distribution for amoxicillin

**Figure S2.** MIC distribution for ampicillin

**Figure S3.** MIC distribution for ceftiofur

**Figure S4.** MIC distribution for cephalothin

**Figure S5.** MIC distribution for colistin

**Figure S6.** MIC distribution for doxytetracycline

**Figure S7.** MIC distribution for enrofloxacin

**Figure S8.** MIC distribution for florfenicol

**Figure S9.** MIC distribution for gentamicin

**Figure S10.** MIC distribution for kanamycin

**Figure S11.** MIC distribution for lincomycin

**Figure S12.** MIC distribution for lincospectin (1:2)

**Figure S13.** MIC distribution for spectinomycin

**Figure S14.** MIC distribution for tiamulin

**Figure S15.** MIC distribution for tilmicosin

**Figure S16.** MIC distribution for tylosin
